# Incorporation of Adeno-Associated Virus Encoding Vascular Endothelial Growth Factor into a Biodegradable Elastomeric Scaffold for Improved Function in the Ischemic Rat Heart

**DOI:** 10.1021/acsbiomaterials.4c01457

**Published:** 2025-02-19

**Authors:** Yasumoto Matsumura, Taro Fujii, Xinzhu Gu, Hong Bin Jiang, Noriyuki Kashiyama, Yasunari Hayashi, Marianna Barbuto, Ying Tang, Bing Wang, Masato Mutsuga, Akihiko Usui, William R. Wagner

**Affiliations:** † Departments of Bioengineering, Surgery and Chemical Engineering, University of Pittsburgh, Pittsburgh, Pennsylvania 15261, United States; ‡ McGowan Institute for Regenerative Medicine, University of Pittsburgh, Pittsburgh, Pennsylvania 15261, United States; § Department of Cardiac Surgery, Nagoya University Graduate School of Medicine, Nagoya, Aichi 466-8550, Japan; ∥ Ri. MED Cardiac Tissue Engineering Laboratory, Ri. MED Foundation, Palermo 90133, Italy; ⊥ Department of Biological, Chemical and Pharmaceutical Science and Technologies, University of Palermo, Palermo 90133, Italy; # Vascular Medicine Institute, Division of Cardiology, Department of Medicine, University of Pittsburgh, Pittsburgh, Pennsylvania 15261, United States

**Keywords:** myocardial infarction, ischemic cardiomyopathy, biodegradable patch, viral vector, AAV, VEGF

## Abstract

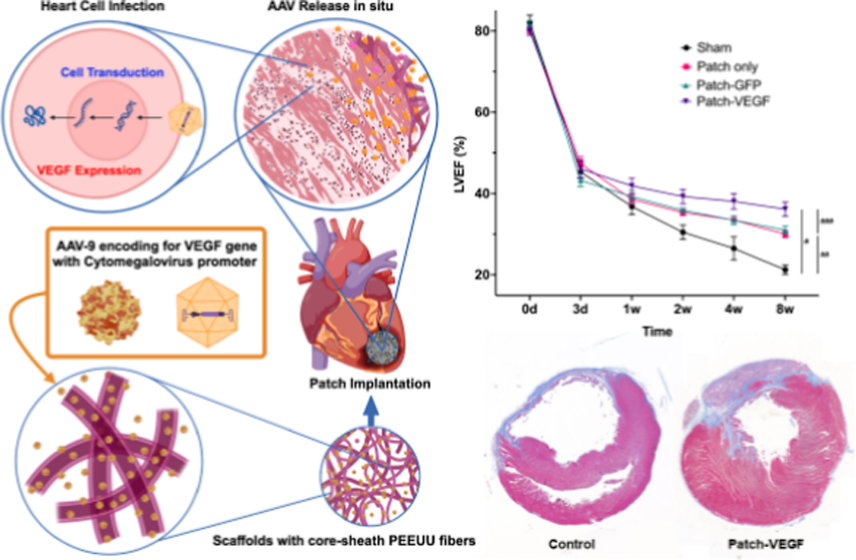

Ischemic heart disease morbidity and mortality ensue as the ventricle remodels, and cardiac function is lost following myocardial infarction. Previous studies have shown that applying a biodegradable, elastic epicardial patch onto the ischemic cardiac wall preserves the cardiac function and alters the remodeling process. In this report, the capacity to deliver a recombinant adeno-associated virus (AAV) encoding human vascular endothelial growth factor (VEGF) was evaluated to determine if it would provide benefit beyond a patch alone. Coaxial electrospinning of a poly(ether ester urethane) urea generated microfibrous patches with fibers loaded in their core with VEGF–AAV in poly(ethylene oxide) or vehicle alone. In a rat infarction model, epicardial patches were placed 3 days post-infarction. Over an 8 week period following the intervention, end-diastolic area was lower and ejection fraction greater in the patch-VEGF group compared with the control patch and sham surgery groups. There was also a greater number of α-SMA-positive cells, blood vessels, and positive immunostaining for VEGF in the patch-VEGF group compared with groups having patches lacking VEGF. The approach of combining mechanical (patch) and biofunctional (controlled release angiogenic therapy) support through a scaffold-based gene vector transfer approach may be an effective option for dealing with the adverse ventricular wall remodeling that leads to end-stage cardiomyopathy.

## 1 Introduction

After myocardial infarction (MI), cardiac function is gradually decreased in concert with left ventricular (LV) dilatation, LV wall thinning, and LV chamber geometry expansion and rounding.^1^ These changes in total, termed LV remodeling, have been associated with the latter stages of progressive heart failure. Pharmacologic treatments applied in this setting include angiotensin converting enzyme inhibitors, angiotensin II receptor blockers (ARB), aldosterone antagonists, diuretics, β-blockers, and antiplatelet therapy. Interventions include cardiac resynchronization therapy, percutaneous coronary intervention, and coronary artery bypass grafting (CABG), which are widely performed in association with ischemic heart diseases (IHD) and have been shown to be highly effective for those patients.^2^ Despite this armamentarium of therapies, IHD remains a progressive and, too often, fatal disease. At the point of end-stage heart failure, the ideal therapy remains cardiac transplantation or, in some patients, mechanical circulatory support as a bridge to transplantation or for chronic support. However, donor organ scarcity and immunosuppression side effects remain a limiting problem.^3,4^

Interrupting or slowing the LV remodeling process and thus potentially halting or slowing cardiac function deterioration to the point of end-stage interventions would be of great value in the care of the post-MI patient. An array of approaches has been reported in the literature in an effort to meet this need. These have included cell therapies, bulking agent injectates, gene therapies, patches, ventricle constraining devices, and pharmacologic agents targeted at remodeling pathways.^5−8^ Among these candidate therapies, we have focused our efforts on the development of a biodegradable, elastic epicardial patch and have shown the benefit of this approach in the porcine model and in various conditions with a rat model.^9−12^ We have also recently demonstrated that it is feasible to create an elastomeric cardiac patch capable of delivering a recombinant adeno-associated virus (AAV) that encodes a gene of interest to the infarcted cardiac wall.

In that earlier study, the extended release and prolonged AAV transgene expression were evaluated compared with direct injection of AAV encoding green fluorescent protein (GFP) and implantation of a poly(ether ester urethane) urea (PEEUU) encoding AAV–GFP patch after cardiac infarction. The details can be found in.^13^ Briefly, PEEUU core-sheath fibrous scaffolds showed an average outer diameter of 15 μm, a core diameter of 10 μm, and a pore size of 40 μm. AAV was conjugated with amine-reactive cyanine 3 (Cy3) for verifying the AAV encapsulated in scaffolds, and the labeled AAV was successfully detected in PEEUU core-sheath fibrous scaffolds. Sustained transgene expression was observed over 8 weeks. In the rat heart, GFP-positive cells colocalized with cardiac troponin- T (cTnT) and α-SMA-positive cells were found to express significantly more GFP in the PEEUU encoding AAV–GFP patch implantation group compared with the AAV–GFP direct injection group. No GFP expression was found in the kidney, lung, liver, and other organs. Sustained and localized viral particle delivery was achieved over 2 months in vitro. Additionally, the timing of mechanical intervention therapy, such as with injectates or patches, was evaluated in a previous study.^14^ Although bulking agent injection therapy, not patch implanting therapy, was used, the results from that study showed that functional outcomes were better if the intervention was performed 3 days after MI compared to immediately or 2 weeks after MI.

The objective of this study was to determine if a similar biodegradable, elastomeric patch loaded with AAV encoding the angiogenic factor, vascular endothelial growth factor (VEGF), would provide structural and functional benefit to the post-MI rat heart and if such an approach would lead to increased local vascularization in the vicinity of the applied epi-cardiac patch. In the previous study, prolonged gene expression was achieved in the patch loaded with AAV encoding GFP. Here, we evaluated the therapeutic potential of AAV encoding the functional gene-VEGF instead of the reporter gene GFP and evaluated changes in cardiac function. The AAV encoding VEGF was suspended in a poly(ethylene oxide) solution and incorporated into a PEEUU patch through a coaxial feed electrospinning technique. The resulting patch material was applied to the epicardium of the ischemic rat left ventricle 3 days after MI and assessed over the following 8 weeks post-implantation.

## 2 Materials and Methods

### 2.1 Polymer Synthesis and Patch Fabrication

Polycaprolactone diol (PCL, Mn = 2000), poly(ethylene glycol) diol (PEG, Mn = 2000), poly(ethylene oxide) (PEO, Mv ∼ 900,000), 1,4-diisocyanatobutane (BDI), putrescine, and tin(II) 2-ethylhexanoate were purchased from Sigma-Aldrich (St. Louis, MO, USA). 1,1,1,6,6,6-Hexafluoroisopropanol (HFIP) was purchased from Oakwood Inc. (Estill, SC, USA). Poly(ether ester urethane) urea (PEEUU) was synthesized from PCL diol, PEG diol, butyl diisocyanate, and putrescine using a 2-step one-pot synthetic strategy.^15^ The stoichiometry of the reaction was 2:1:1 BDI/(PCL + PEG): putrescine. PEEUU designates polyurethane urea consisting of PCL and PEG blocks with a feed molar ratio of 75:25. The detailed synthesis and characterization of PEEUU can be found in an earlier report.^13^

**Figure 1:**
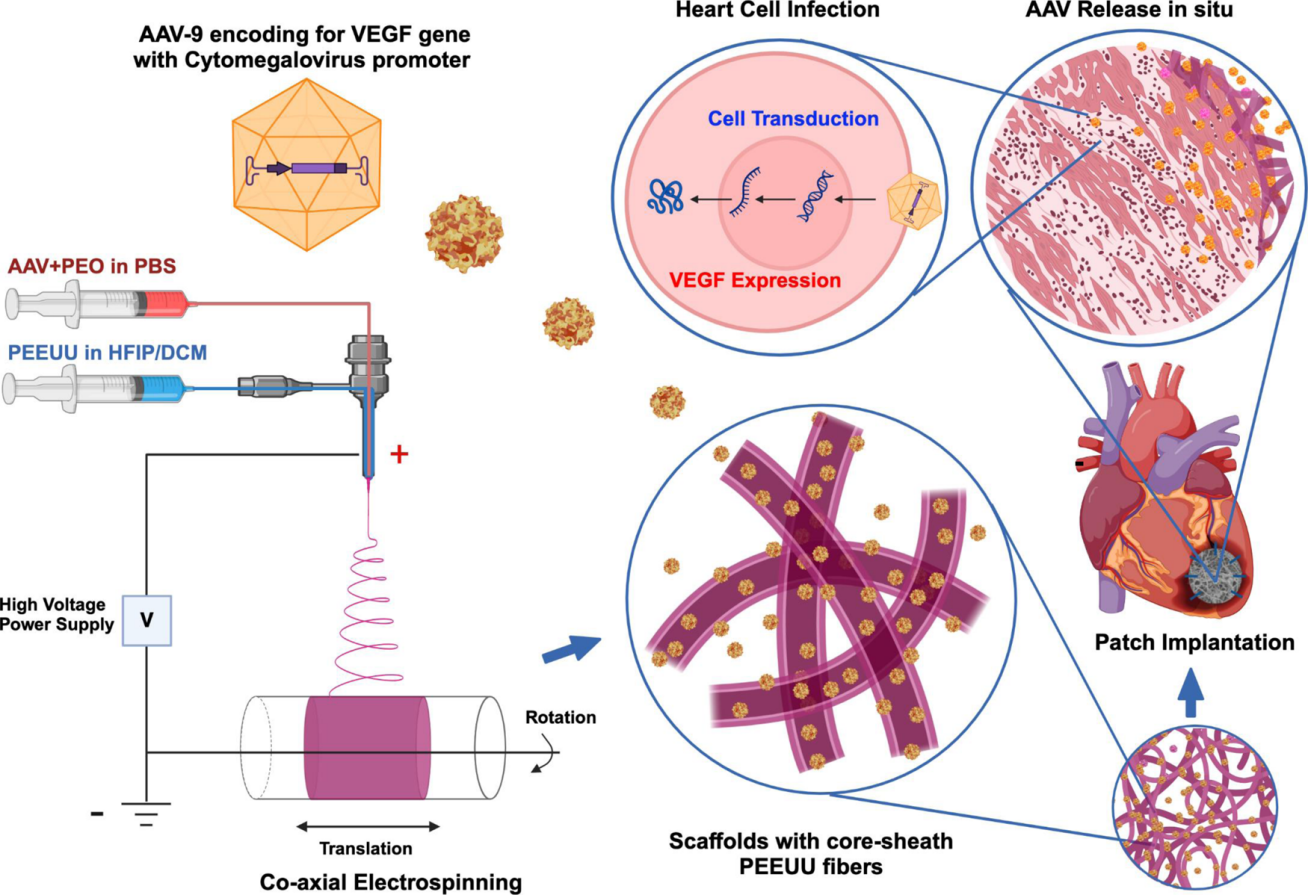
Schematic overview of this study.

**Figure 2:**
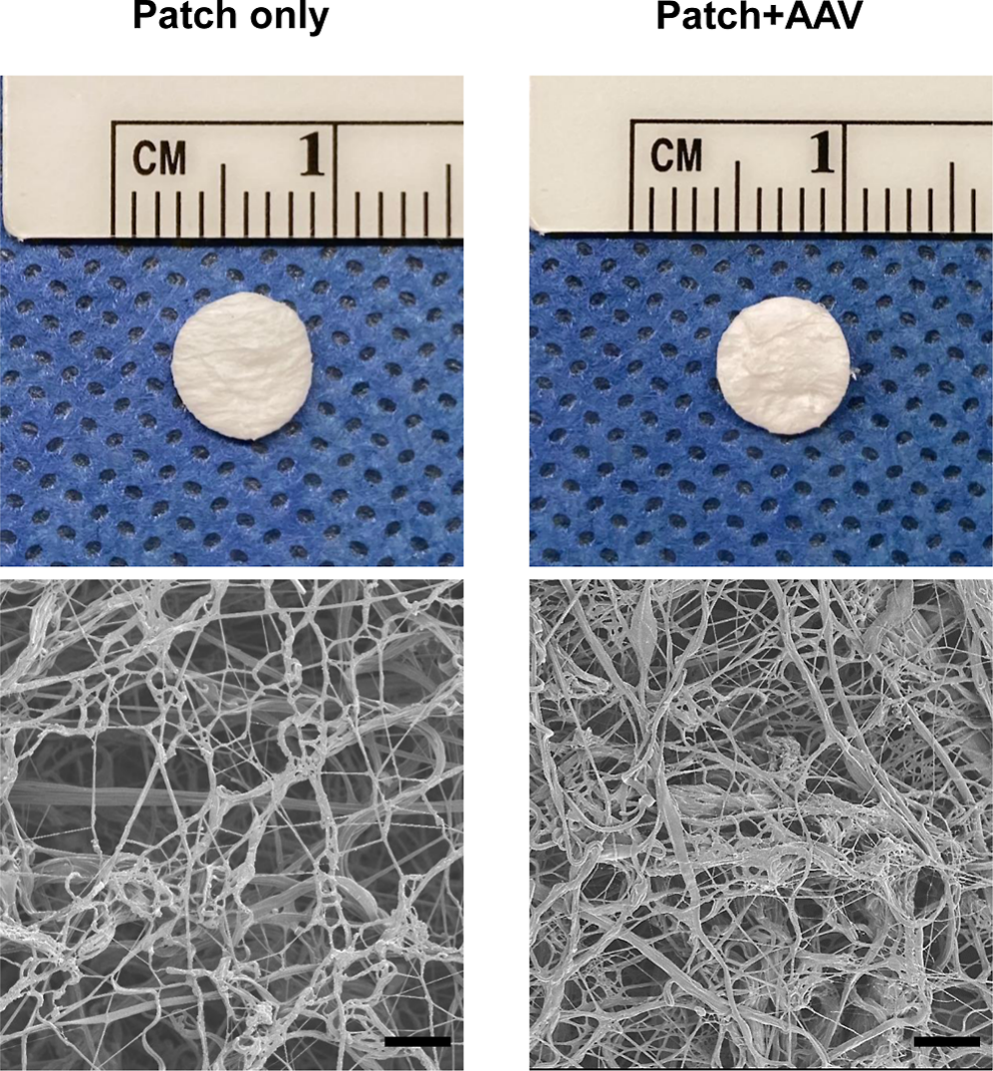
Scaffolds with a 6 mm diameter (upper) and electron micrographs (lower). Scale bars = 20 μm.

Scaffolds with core-sheath fibers were made by coaxial electrospinning where AAV9 cmv–VEGF (2.5 × 10^12^ GC/mL) was mixed in 1% PEO in PBS as the core feed solution and PEEUU solution (12 w/v % in 70:30 ratio of DCM/HFIP) as the feed for the sheath. Flow rates for core and sheath solutions were set at 0.6 and 1.5 mL/h, respectively. Electrospun fibers were collected onto a rotating and translating stainless steel mandrel located 17 cm from the tip of the discharging capillary to a thickness of 100 μm. The voltage difference between the capillary and mandrel was 19 kV (Figures 1 and 2).

### 2.2 Vector Construction and Transduction In Vitro

Human VEGF gene was carried by a self-complementary AAV vector and driven by the CMV promoter. AAV9 viruses were made according to a three-plasmid cotransfection method and purified through CsCl density gradient ultracentrifugation, as per published protocols.^16^ Vector titers were determined by the DNA dot blot method and were approximately 5 × 10^12^ ∼ 1 × 10^13^ viral particles per mL.^17^ The effectiveness of AAV9 cmv–VEGF was tested by transduced HEK293 cells. HEK 293 cells (passage 34–38) were cultured in Dulbecco’s modified Eagle’s medium (DMEM) supplemented with 10% fetal bovine serum and 1% penicillin/streptomycin (P/S). A monolayer of 10^4^ cells was cultured at the bottom of 12-well plates. AAV with different titers (high titer 2.5 × 10^11^ and low titer 2.5 × 10^10^) were dropped in each well, respectively. Cells were cultured for an additional 3 days. Then the level of VEGF expression in the supernatants was quantified using Human VEGF Quantikine ELISA Kit (R&D Systems, Minneapolis, MN, USA). Cells without AAV and DMEM alone were used as controls (Figure S1).

### 2.3 Animal Procedures

#### 2.3.1 Experimental Groups

Adult female syngeneic Lewis rats (10–12 weeks old, Harlan Sprague Dawley Inc., Indianapolis, IN, USA) weighing 180–220 g were used in this study. For handling ease over the experimental period, only female rats were studied due to their slower growth.^18^ The research protocol followed the National Institutes of Health guidelines for animal care and was approved by the Institutional Animal Care and Use Committee of the University of Pittsburgh. Rats undergoing the MI procedure were randomly divided into three groups: (1) control group with sham patch placement surgery, (2) PEEUU patch made with core-sheath fiber patch having a core of PEO but no virus as the patch control group, and (3) PEEUU with rAAV9 encoding VEGF loaded into the PEO core material as the patch-VEGF group.

#### 2.3.2 Anesthesia and Analgesia

All surgical procedures were performed under general anesthesia. Rats were anesthetized with 3.0% isoflurane inhalation with 100% oxygen. Each rat was orally intubated and mechanically supported by a rodent volume-controlled mechanical ventilator (683 Ventilator, Harvard Apparatus, Holliston, MA, USA) at a tidal volume of 3 mL and 80 breaths/min. 0.1 mg/kg of buprenorphine was injected twice a day for 3 days after procedures for analgesic treatment.

#### 2.3.3 MI Procedure

MI was created by ligation of the proximal left anterior descending artery (LAD) as previously described.^19^ Briefly, the rat was placed on a warming pad in a supine position under general anesthesia. The chest was shaved and sterilized with a povidone-iodine solution. Procedures were performed in a sterile environment. The heart was exposed through the fourth left thoracotomy, and the proximal LAD was directly ligated with 5–0 polypropylene. MI was confirmed by monitoring decreased motion of the cardiac wall, and the incision was closed in layers. 10 mg/kg of lidocaine was injected intramuscularly before and after coronary ligation to prevent fatal arrhythmia. After fully recovering from anesthesia, the animals were extubated and returned to their cages. 20 mg/kg cefazolin was injected twice a day for 3 days after procedures to prevent surgical site infection.

#### 2.3.4 Patch Implantation

Patches were generated from a scaffold sheet with a 6 mm diameter surgical punch, which was large enough to cover the entire infarction area (Figure 2). The implantation procedures were performed 3 days after MI creation as previously described.^12^ Briefly, the heart was exposed with the same incision through the fifth thoracotomy under general anesthesia. The surface of the infarcted cardiac wall was lightly scraped to introduce blood into the patch and create adhesion to the patch. The patch was implanted with a continuous running suture with 7–0 polypropylene to cover the entire infarcted region. For the control group, the surface of the infarcted cardiac wall was just scraped without implanting the patch. The incision was closed in layers.

#### 2.3.5 Echocardiography

Cardiac functions were evaluated with echocardiography before surgery, 3 days after MI, and 1, 2, 4, and 8 weeks after implantation therapy. Echocardiography was performed using an Acuson Sequoia C256 system with a 13 MHz liner ultrasonic transducer (15L8; Acuson Corporation, Mountain View, CA, USA) with 1.5% isoflurane inhalation with 100% oxygen without mechanical ventilation. Rats with infarcts smaller than 25% of the LV free wall at 3 days after MI were excluded in this study. The end-diastolic area (EDA), end-systolic area (ESA), end-diastolic dimension (LVDd), and end-systolic dimension (LVDs) were obtained from short axis views at the level of the LV papillary muscle using NIH image J software. The fractional area change (%FAC) and fractional shortening (% FS) were calculated as % FAC = (EDA–ESA)/EDA × 100% and % FS = (LVDd–LVDs)/LVDd × 100%, respectively. Ventricular volume (V) was estimated using the formula of Teicholz as follows to calculate LV end-diastolic volume (LVEDV) and end-systolic volume (LVESV), V = 7.0/(2.4 + *D*) × *D*^3^. *D* is the diameter of LV measured by M-mode echocardiography. LV ejection fraction (LVEF) was calculated as LVEF = (LVEDV–LVESV)/LVEDV × 100%.^20^ For the analysis of echocardiographic parameters, a second control group (patch-GFP) was included from the earlier study,^13^ where the same model was implemented and the same parameters measured. This historic data provide a comparison to a scaffold incorporating AAV, but with GFP instead of a functional gene.

#### 2.3.6 Post-Mortem Study

At 8 weeks post-patch implantation or control procedure, the animals were euthanized by directly injecting potassium chloride into the LV to prevent the collapse of cardiac vessels, and the hearts were dissected at the center of ischemic area (center of the patch) along the longitudinal axis. Samples were fixed with 10% formalin and stained with Masson’s trichrome or labeled with antibodies against alpha smooth muscle actin (α-SMA) (ab7817, Abcam, Cambridge, MA, USA), CD31 (ab28364, Abcam, Cambridge, MA, USA), lectin galactoside-binding soluble 3 (MAC2) (ARP54688_P050, Aviva system biology, San Diego, CA, USA), and VEGF (ab52917, Abcam, Cambridge, MA, USA), respectively. Samples were roughly divided into five sections, and high magnification fields were captured in each animal and used to calculate the average number of cells or vessels. Vessels were defined as α-SMA or CD31-positive structures having a visible lumen and a diameter of more than 10 μm. Images were collected both in the area of the patch and in the peri-patch region. NIH image J software was used for the analysis.

### 2.4 Statistics

Differences between the two groups were tested using the Student’s *t*-test. One-way repeated measures analysis of variance followed by Tukey’s test was applied for multigroup comparisons in echocardiography analysis. All statistical analyses were conducted using GraphPad Prism for Mac (Version 8, San Diego, CA, USA). Data are expressed as mean ± standard error of the mean. *P*-values <0.05 were considered significantly different.

## 3 Results

### 3.1 Postoperative Course

A total of 25 rats were utilized in this study. Two rats died during the first surgery due to fatal arrhythmia, and 1 was excluded because of small infarction (less than 20% of the LV free wall involved). The remaining 22 rats were randomly divided into the 3 groups (7 in control, 7 in patch, and 8 in patch-VEGF groups). There was 1 death at 24 h after the second surgery because of cardiac tamponade due to bleeding (patch-VEGF group), and 7 rats completed the study in each group. At the time of sacrifice, the patch was observed to almost fully cover the infarcted zone in all cases.

### 3.2 AAV–VEGF-Loaded Patch Improves the Functionality of the Ischemic Rat Heart

**Figure 3:**
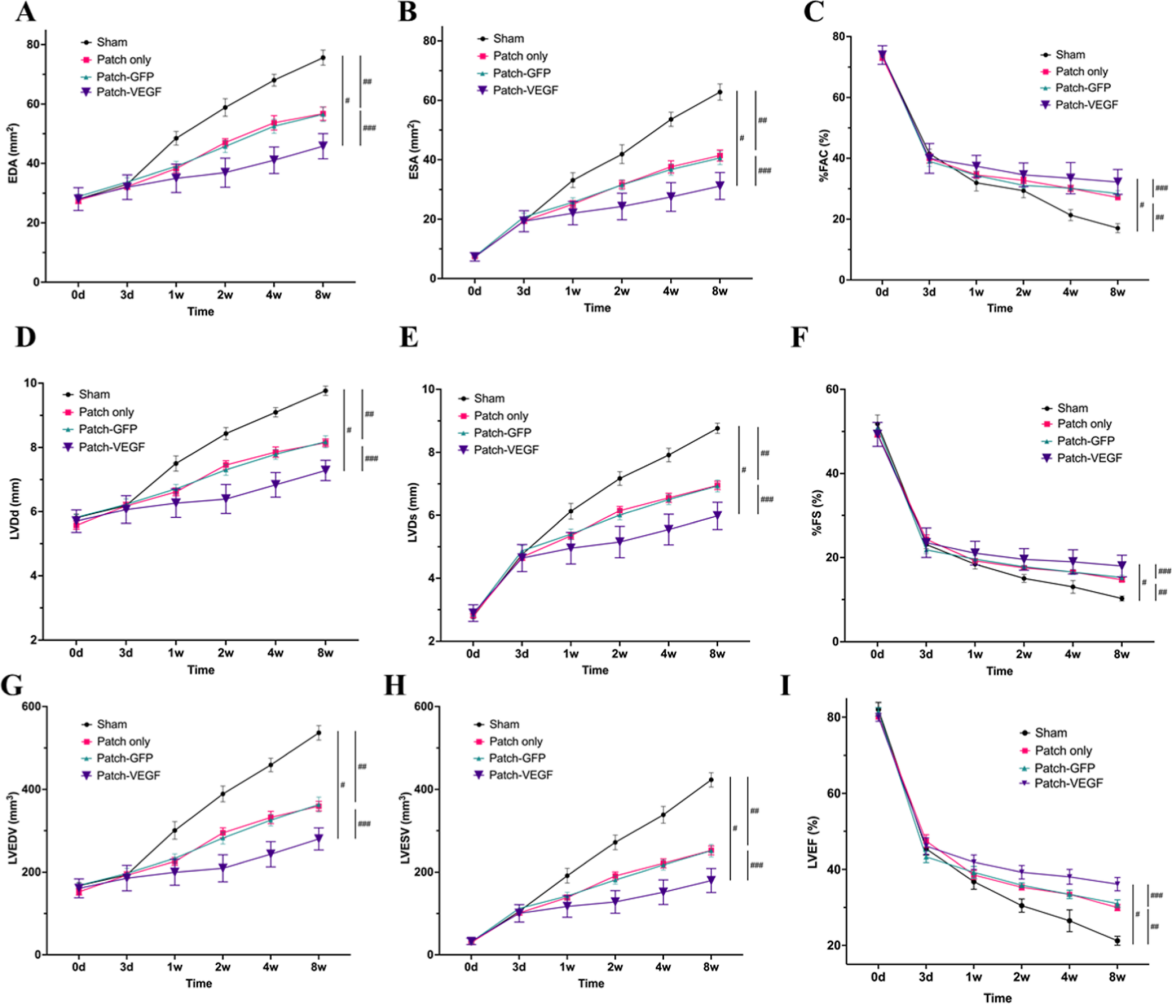
Echocardiography assessment up to 8 weeks after treatment. A second control group (patch-GFP) from an earlier study in the same model was included for comparison purposes.^13^ (A) EDA, (B) ESA, (C) % FAC, (D) left ventricular end-diastolic diameter (LVDd), (E) left ventricular end-systolic diameter (LVDs), (F) % FS, (G) LVEDV, (H) LVESV, and (I) LVEF. #, ##, and ###: significant difference between sham and patch-VEGF groups, sham and patch only groups, and patch only and patch-VEGF groups, respectively. No significant difference detected between patch only and patch-GFP groups. *P* < 0.05. *N* = 7 in control, 7 in patch, and 8 in patch-VEGF groups.

Transthoracic echocardiography revealed no significant differences prior to MI treatment in all groups. At the 8 week time point after treatment, EDA and ESA were significantly smaller, and % FAC was significantly greater for the patch-VEGF group compared with the patch and control groups (EDA: 45.8 ± 1.5 vs 56.8 ± 2.1 vs 75.6 ± 2.3 mm^2^, ESA: 31.2 ± 1.6 vs 41.4 ± 1.7 vs 62.8 ± 2.5 mm^2^, %FAC: 32.2 ± 1.4 vs 27.1 ± 0.4 vs 17.1 ± 1.4%, patch-VEGF vs patch vs control, *p* < 0.03, respectively) (Figure 3A–C). LVDd and LVDs were significantly smaller, and %FS was significantly greater for the patch-VEGF group compared with the patch and control groups (LVDd: 7.34 ± 0.11 vs 8.15 ± 0.11 vs 9.76 ± 0.13 mm, LVDs: 6.06 ± 0.15 vs 6.95 ± 0.12 vs 8.76 ± 0.15 mm, % FS: 18.0 ± 0.9 vs 14.7 ± 0.4 vs 10.3 ± 0.6%, patch-VEGF vs patch vs control, *p* < 0.03, respectively) (Figure 3D–F). Moreover, LVEDV and LVESV were significantly smaller, and LVEF was significantly greater for the patch-VEGF group than the other groups (LVEDV: 280 ± 9 vs 359 ± 10 vs 537 ± 16 mm^3^, LVESV: 180 ± 10 vs 252 ± 10 vs 423 ± 16 mm^3^, LVEF: 36.2 ± 1.6 vs 30.0 ± 0.8 vs 21.2 ± 1.1%, patch-VEGF vs patch vs control, *p* < 0.03, respectively) (Figure 3G–I). To allow comparison to a relevant historical control and avoid unnecessarily replicating an animal experiment, data were included in Figure 3 for echocardiographic analysis of a PEEUU patch encoding AAV–GFP applied in an identical rat MI model and reported earlier.^13^ This group represented a control for all variables except the encoded gene and is termed the patch-GFP group. No significant differences were detected between the patch and patch-GFP groups.

### 3.3 AAV–VEGF-Loaded Patch Enhances Angiogenesis in the Ischemic Rat Heart

**Figure 4:**
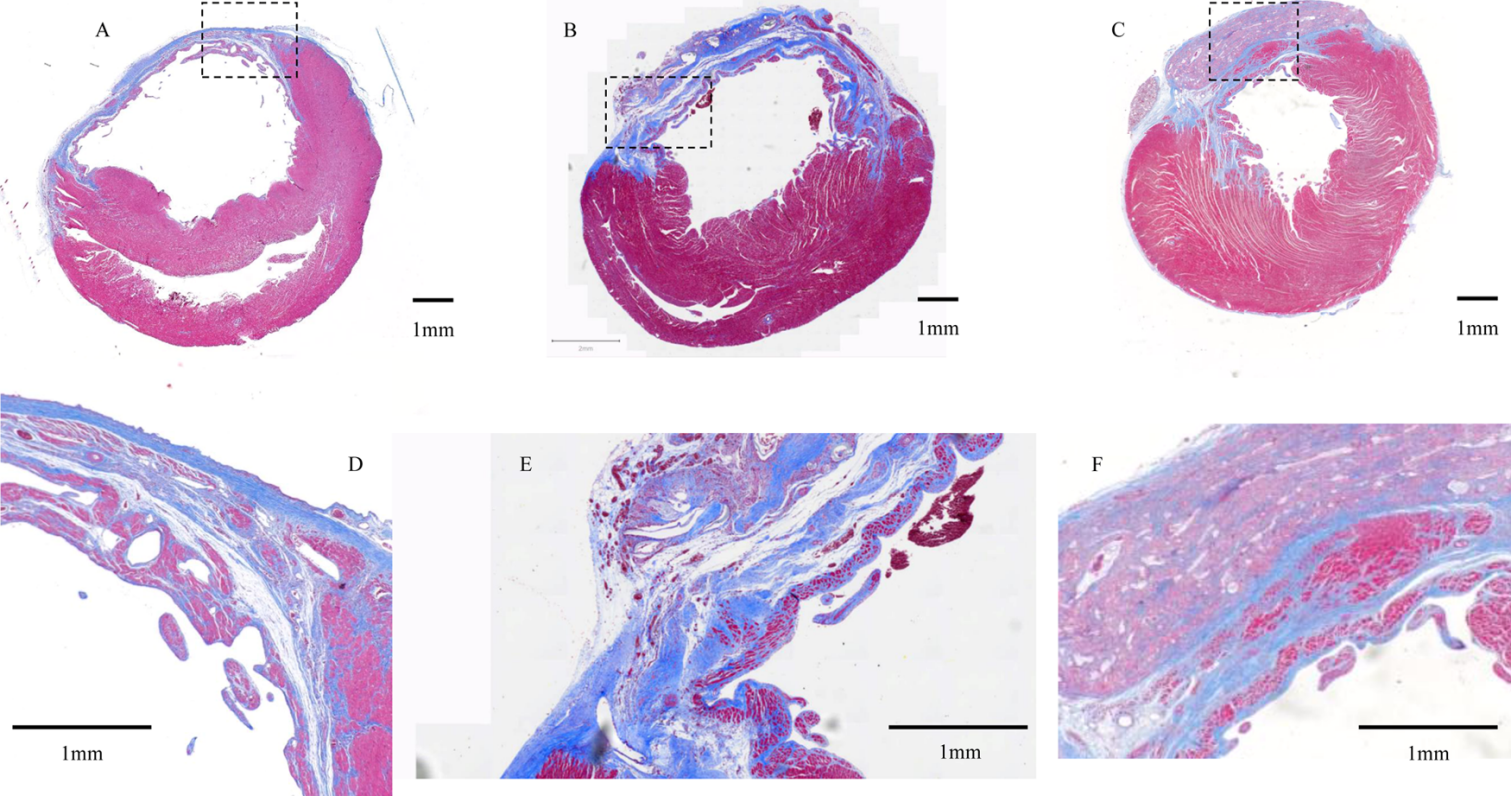
Masson’s trichrome staining for cardiac sections at 8 weeks following patch implantation. (A) Control, (B) patch only group, and (C) patch-VEGF group. Dashed box indicates higher magnification area ((D–F) corresponding to (A–C) groups, respectively). Yellow lines indicate the patch.

**Figure 5:**
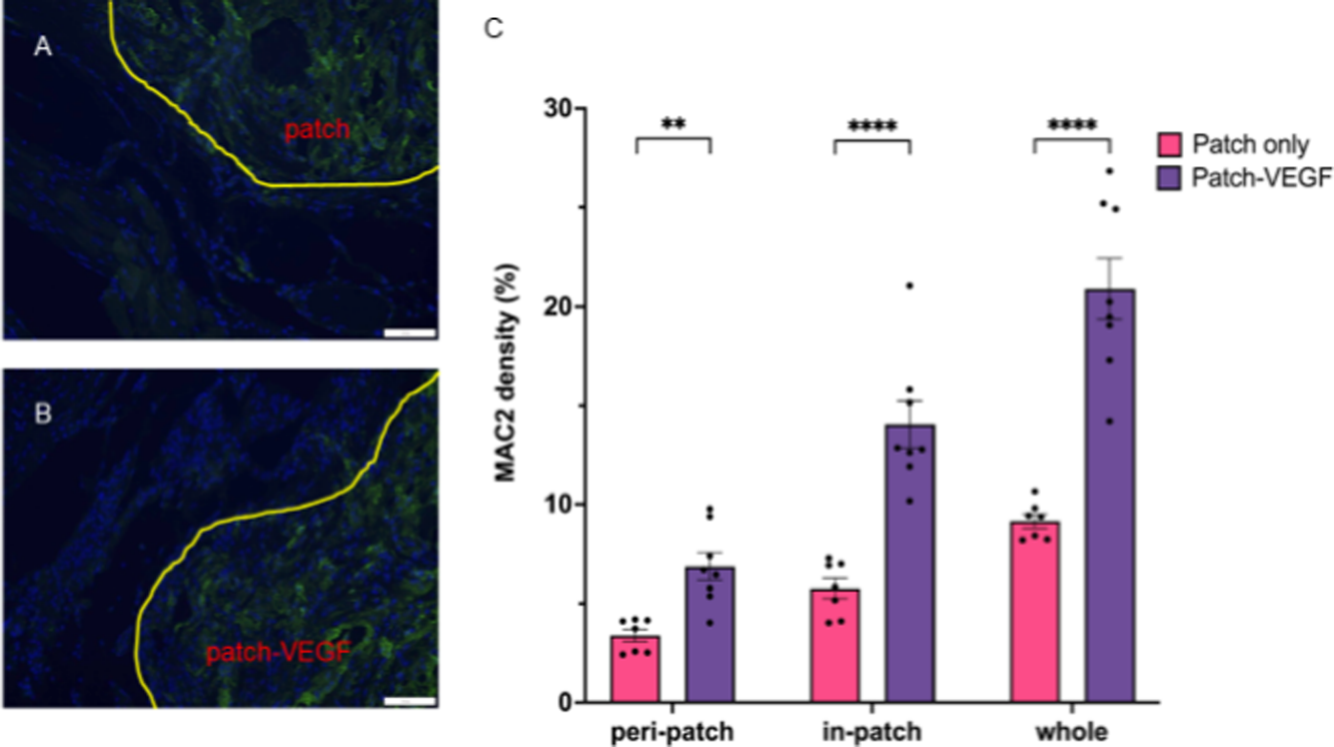
Immunofluorescence staining with DAPI and anti-MAC2. Yellow lines indicate the border between heart tissue and the patch. (A) Patch only group; (B) patch-VEGF group. (C) MAC2-positive area counted by ImageJ software. **p < 0.01; ****p < 0.0001. Scale bars = 100 μm. *N* = 7 in patch and 8 in patch-VEGF groups.

**Figure 6:**
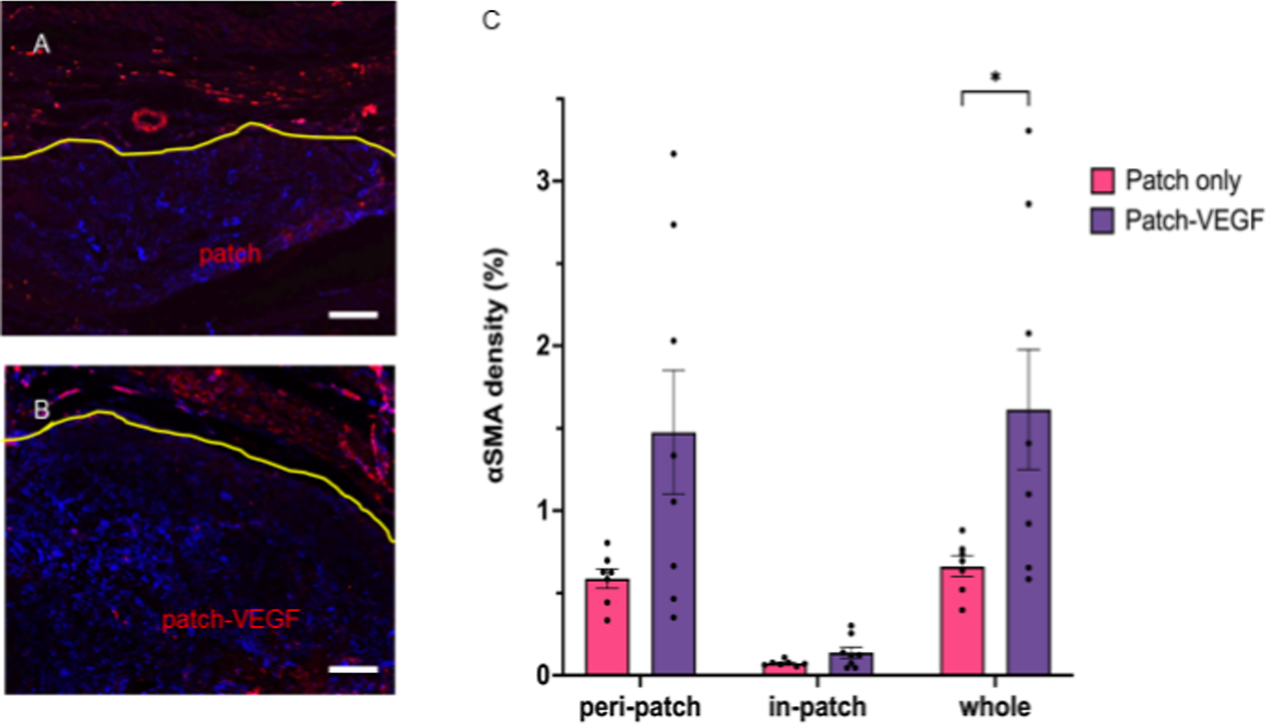
Immunofluorescence staining with DAPI and anti-αSMA. Yellow lines indicate the border between heart tissue and the patch. (A) Patch only group; (B) patch-VEGF group. (C) αSMA-positive area counted by ImageJ software. *p < 0.05. Scale bars = 100 μm. *N* = 7 in patch and 8 in patch-VEGF groups.

**Figure 7:**
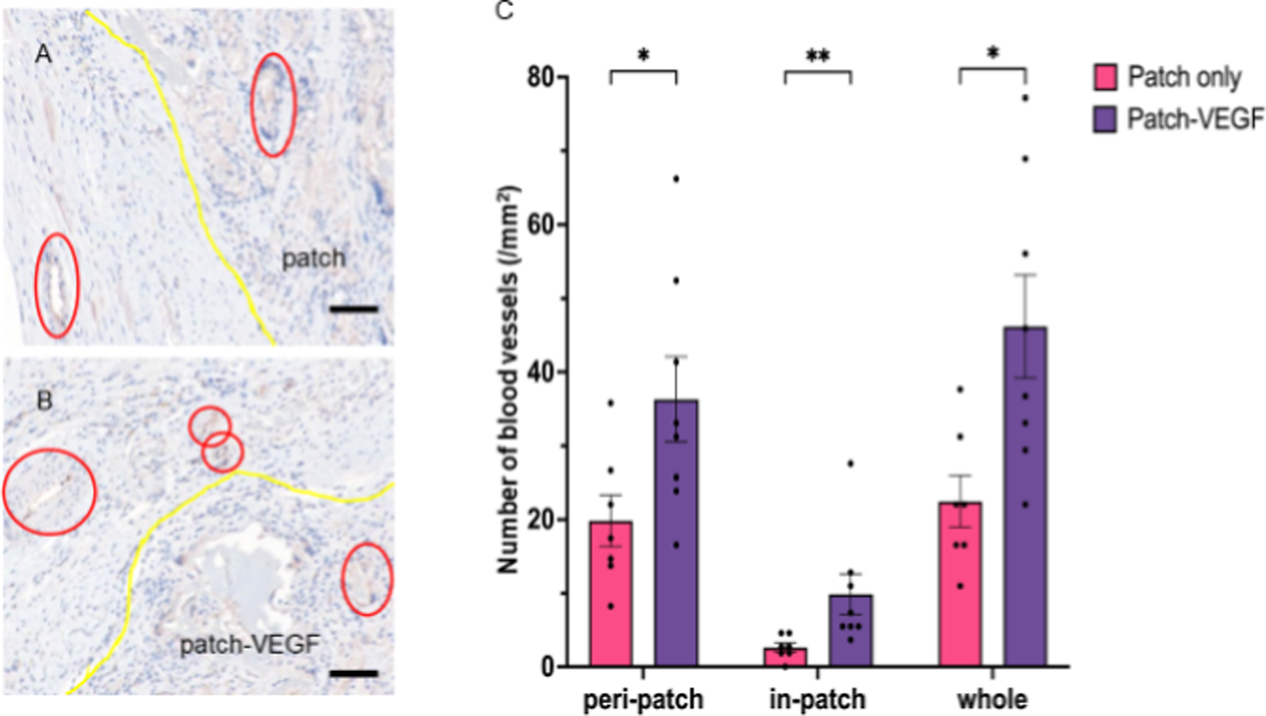
DAB staining with anti-CD31. Yellow lines indicate the border between heart tissue and the patch. Red circles indicate CD31-positive vessels. (A) Patch only group; (B) patch-VEGF group. (C) Number of CD31-positive vessels. *p < 0.05; **p < 0.01. Scale bars = 100 μm. *N* = 7 in patch and 8 in patch-VEGF groups.

**Figure 8:**
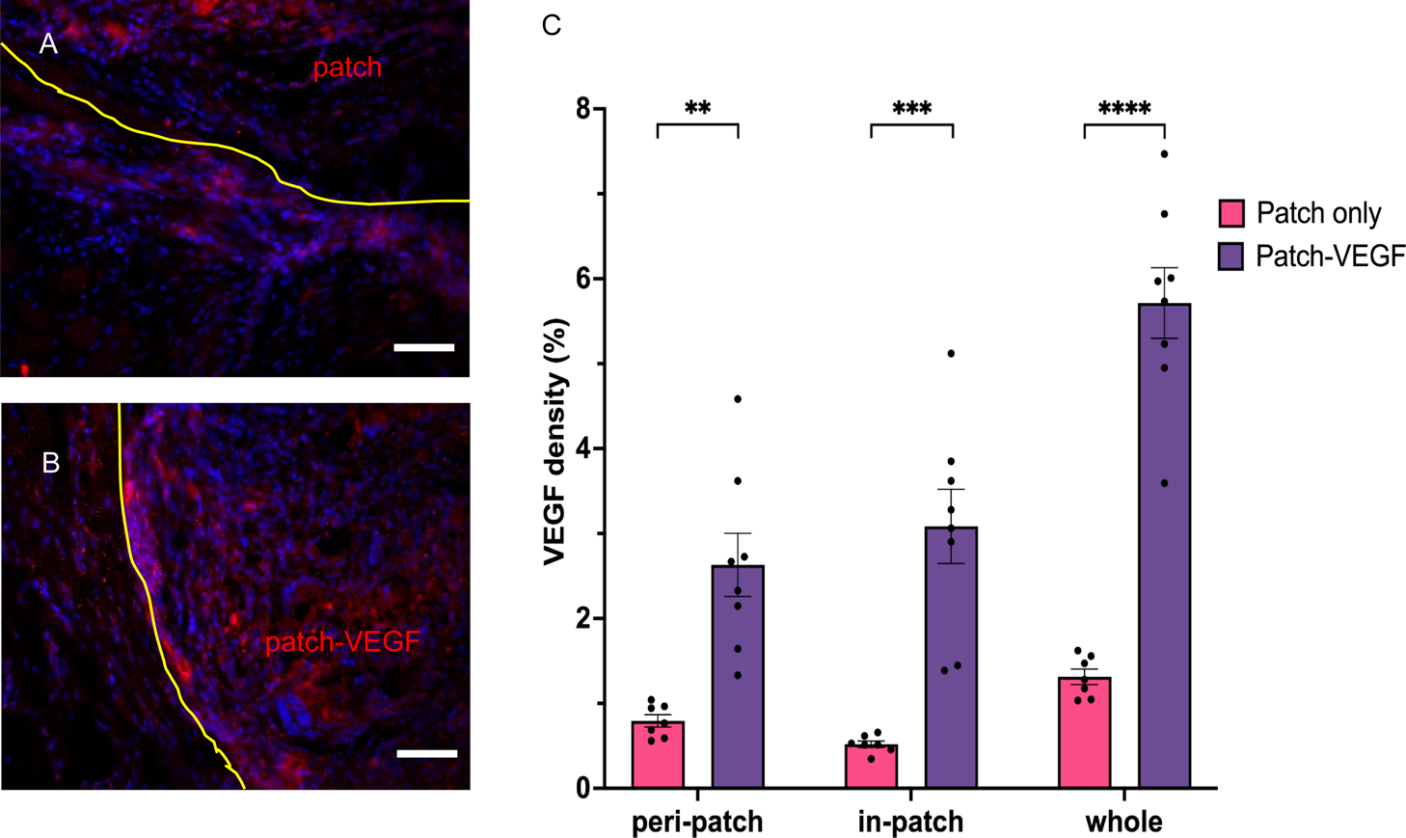
Immunofluorescence staining with DAPI and anti-VEGF. Yellow lines indicate the border between heart tissue and the patch. (A) Patch only group; (B) patch-VEGF group. (C) VEGF-positive area counted using ImageJ software. **p < 0.01, ***p < 0.001, and ****p < 0.0001. Scale bars = 100 μm. *N* = 7 in patch and 8 in patch-VEGF groups.

Samples were obtained from all of the groups after euthanasia. As can be determined in Figure 4, the patches containing AAV–VEGF or patch alone were located over the ischemic LV wall and experienced cellular infiltration similar to that seen for earlier studies.^13^ Looking at the cellular infiltrate characteristics in the two groups, a greater number of MAC2-stained cells were detected in the patch-VEGF group compared to the patch control group (Figure 5). The results of α-SMA and CD31 staining are shown in Figures 6 and 7. A greater number of α-SMA-positive cells were detected in the patch-VEGF group, and also the number of vessels with more than a 10 μm lumen was higher in the patch-VEGF group. Although these vessels were mainly detected around the border zone between the patch and the ischemic wall, some vessels appeared to have migrated into the patch. Figure 8 shows the VEGF staining densities for the two patch groups. VEGF was detected to a greater extent in the patch carrying VEGF encoding AAV, and a greater number of VEGF-positive cells were detected in the peri-patch regions with the ischemic wall.

## 4 Discussion

Gene therapy for ischemic heart disease has been a focus of investigation for several decades and continues to offer a mechanism through which specific LV remodeling pathways might be altered for improved functional outcomes. AAV is one of the most studied viral vectors and is attractive in that AAV is a small, single-stranded DNA virus and offers the potential for longer term transgenic expression.^21^ There are many serotypes of AAV, such as AAV1, AAV2, AAV6, AAV8, AAV9, and others, that have been identified and evaluated for their ability to facilitate gene expression in the heart. Among these serotypes, AAV9 is considered to be the most efficient vector for ischemic heart disease.^22−29^ To deliver AAV to the target tissue, the following methods have been considered: intravenous injection, ante/retrograde coronary infusion, and direct injection into the ischemic LV wall.^30−34^ Systemic injection provides an easy means of AAV delivery, but it remains limited to small animal studies due to the inefficiency of this approach in targeting the desired tissue.^35^ In a recent study, Wu C. et al. reported injecting at the time of MI a calcium-cross-linked alginate hydrogel incorporating AAV9-VEGF and lignosulfonate-doped polyaniline (PANI/LS) nanorods for conductance to positively impact cardiac remodeling and preserve cardiac function.^36^ Similar to our report, AAV–VEGF was incorporated into the hydrogel injectate for prolonged gene expression, whereas our effort combined the gene delivery from an epicardial-placed, mechanically supportive PEEUU patch. In a clinical sense, the timing of the intervention may be important as injection at the time of MI may not be feasible. In a survey from a nationwide database in the US, Elbadawi et al. reported higher mortality of isolated CABG for MI patient on day one compared with day three or more;^37^ also, in an earlier study from our group with a similar rat MI model, it was shown that beneficial effects from passive injectates were much improved at 3 days post-MI^14^ versus earlier or later injection times. However, the optimal time for patch implantation remains an open question for further study.

There have been multiple clinical trials of gene therapy for HF using coronary infusion delivery. The result of the calcium upregulation by percutaneous administration of gene therapy in patients with cardiac disease (CUPID) trial was published in 2016 as the largest gene transfer study for the treatment of HF.^38^ CUPID 2 was a phase 2b trial using AAV1 vector encoding sarcoplasmic/endoplasmic reticulum Ca^2+^-ATPase (SERCA2a) for HF patients via anterograde coronary infusion. 250 patients aged 18 to 80 with moderate-severe HF at 67 clinical centers participated in this randomized, multinational, double blind, placebo-controlled study. Unexpectedly, the result of CUPID 2 showed that AAV1 encoding SERCA2a did not reduce either recurrent heart failure or terminal events. The stromal cell-derived factor-1 (SDF-1) plasmid treatment for patients with heart failure (STOP-HF) trial is a phase 2, double blind, randomized, placebo-controlled trial for treatment of HF via endomyocardial injection.^39^ Unlike the CUPID trial or the current study, a plasmid vector instead of viral vector was used in the STOP-HF trial. Although statistical differences were not detected for the primary end point, the improvement of LVEF in a subset of low EF (<26%) patients was a remarkable result.

Although no safety issues or worsening of clinical course were detected in either of the above trials, the primary outcomes were not satisfied. One of the reasons why these therapies did not show effectiveness for HF might be the duration of gene expression. For intracoronary perfusion, the coronary flow is limited, especially in chronic occlusion, even after coronary intervention, since the vascular bed is reduced in the damaged myocardium. Also, the venous drainage would act to transport the delivered gene immediately after the perfusion. For myocardial injection, the risk of leakage of the injected gene from the injection site is unavoidable and unpredictable. In our previous report, we implanted a PEEUU patch incorporating AAV encoding GFP onto the ischemic LV wall 3 days following MI and evaluated the gene expression compared with direct injection of AAV–GFP.^13^ Much greater levels of gene expression were observed in the patch group versus the injection group 12 weeks after implantation. Based on the potential displayed in those results, we used AAV–VEGF, instead of the AAV–GFP used in the prior study, as a therapeutic gene and demonstrated a higher density of VEGF protein in and around the AAV–VEGF patch at 8 weeks post-implantation. This result suggested that the AAV–VEGF remained in the patch for an extended period, and prolonged gene expression was induced in the ischemic region. The extended release behavior with a functionally beneficial gene that was demonstrated by this patch opens the possibility of combining a mechanically supportive patch with effective gene therapy. Such an approach would provide a multilevel interruption strategy to counteract ventricular remodeling in ischemic cardiomyopathy.

Using locally placed microspheres may be another option for delivering VEGF to the heart.^32^ Rodness et al. showed that sustained delivery of VEGF was obtained by implantation of a VEGF-loaded microsphere patch covered with a chitosan sheet onto the ischemic rat heart. LV function was sustained, and the VEGF-loaded patch still remained at 4 weeks after implantation. Although the concepts in that report of applying passive mechanical support and sustained VEGF exposure are similar, relatively large amounts of material were needed to be placed onto the heart for prolonged protein release. Different from the current study, the VEGF protein may be susceptible to in situ degradation post-implantation, and mechanical support might differ with a hydrogel material that would be expected to have a lower modulus and elasticity than the biodegradable patch of the current report.

There were some limitations of this study worth noting. First, the duration of 8 weeks following treatment is relatively short to investigate the chronic effectiveness of the therapy. Although cardiac function was sustained compared to that of the control groups, there were also no adverse events that occurred during the study period. An extended study duration might be able to demonstrate higher early death rates, where cardiac function continues to deteriorate. AAV–VEGF incorporated into the PEEUU patch achieved VEGF expression 8 weeks after implantation, but elevated VEGF expression in off-target tissues was not evaluated and could be a confounding problem if it occurs. Even though the controlled delivery of virus from the patch is theoretically more restricted to the ischemic wall than other methods such as intravenous or intracoronary infusion, safety evaluation should be assessed over a longer time period. Also, the loading dose of the vector could be a consideration since a single dose of vector was evaluated in this study. Based on the previous study,^13^ we hypothesized that AAV–VEGF would exhibit controlled release behavior similar to AAV–GFP in the same setting. Groups with controlled injection(s) of AAV–VEGF were not assessed as controls in this study. Direct injection of AAV–VEGF into the ischemic or border zone cardiac tissue group would be needed to demonstrate prolonged gene expression following patch implantation with delivery of AAV–VEGF, since AAV–VEGF delivery behavior may vary from AAV–GFP (although both in vivo studies utilized the AAV9 serotype). In terms of histological analysis, further analyses may be warranted in the future. For assessing the inflammatory response, samples were labeled with MAC2 as a pan-macrophage marker, but macrophage phenotypes and other inflammatory markers were not assessed. Although α-SMA expression is not commonly found in adult cardiac tissue but in embryonic myocardium, in our previous study,^10^ we found α-SMA expression in the ischemic cardiac wall beside the patch with coexpression of sarcomeric α-actinin (α-actinin) and cTnT. α-SMA-expressing cells could be further evaluated with costaining of α-SMA and cTnT and/or α-actinin to explore their potential lineage. Finally, this study utilized a small animal model. Although AAV–VEGF patch treatment showed significant effectiveness in this study, it may not directly translate to larger animals with thicker cardiac walls or to future clinical studies. We should consider several parameters before advancing to a large animal (e.g., porcine or ovine) study such as the size of the patch and the timing of implantation. With the larger animal model, cardiac computed tomography or magnetic resonance imaging may be more useful and objective to assess the cardiac function and geometry than echocardiography; also, positron emission tomography could be applied for assessing the metabolism. To demonstrate the formation of functional vessels, perfusion imaging would be of value. Other cardiac data, such as cardiac index or strain parameters, adding circulating levers of brain natriuretic peptide or *N*-terminal pro-BNP will be helpful for assessing the progression of heart failure. Additionally, measuring the level of cardiac-TnT and/or CK-MB could be performed to assess ischemic damage of cardiac muscle. Because the patch is sutured onto the cardiac surface, it is possible that coronary blood flow might be adversely affected, and local infarction may occur. Even though patch implantation has been shown to improve cardiac remodeling, assessment of these markers to exclude the potential damage of patch implantation would be needed. For small animals, it is attractive to use echocardiography because of their size and higher heart rate. Adding these data will be helpful to better assess cardiac function in future large animal studies.

## 5 Conclusions

In this study, an AAV encoding VEGF was coaxially incorporated into the microfibers of a PEEUU patch that was implanted onto the ischemic LV wall 3 days following MI in a rat model. Comparisons of this therapy were made to control (sham surgery) and patch alone groups, as well as historic data with the same type of patch loaded with an AAV encoding GFP. Cardiac function was significantly greater, and more vascularization was found in the rat hearts where the VEGF encoding patch was placed. The patch alone group showed better outcomes than the sham control group, attributable to the provided mechanical support, as shown in earlier studies. Additionally, the patch-VEGF group showed better function compared with the patch alone and patch-GFP groups, attributable to sustained VEGF gene expression during the study period. The concept demonstrated here, of a gene-delivering scaffold combining mechanical support with controlled release of gene encoding a beneficial factor, may offer an improved option for dealing with the adverse ventricular wall remodeling that leads to end-stage cardiomyopathy.

## Supporting Information Available

In vitro transduction of HEK293 cells by AAV9 cmv-VEGF quantified by ELISA (PDF)
(PDF)
