# Supplementary material for: Incorporation of Adeno-Associated Virus Encoding Vascular Endothelial Growth Factor into a Biodegradable Elastomeric Scaffold for Improved Function in the Ischemic Rat Heart
Source: ACS Biomater Sci Eng. 2025 Feb 19;11(4):2226–35. doi: 10.1021/acsbiomaterials.4c01457 (PMC12001208; doi:10.1021/acsbiomaterials.4c01457)
Supplement: Supplementary file 2 — ab4c01457_si_002.pdf [file ab4c01457_si_002.pdf]

Incorporation of adeno-associated virus (AAV) encoding vascular endothelial growth factor (VEGF) into a biodegradable elastomeric scaffold for improved function in the ischemic rat heart

Yasumoto Matsumura[1,2,3], Taro Fujii[1,2,3], Xinzhu Gu[1,2], Jiang Hongbin[1,2], Noriyuki Kashiyama[1,2], Yasunari Hayashi[1,2,3], Marianna Barbuto[1,2,4,5], Ying Tang[6], Bing Wang[1,2,6], Masato Mutsuga[3], Akihiko Usui[3], William R. Wagner[1,2*]

1. Departments of Bioengineering, Surgery and Chemical Engineering, University of Pittsburgh, Pittsburgh, PA, US
2. McGowan Institute for Regenerative Medicine, University of Pittsburgh, Pittsburgh, PA, US
3. Department of Cardiac Surgery, Nagoya University Graduate School of Medicine, Nagoya, Aichi, JP
4. Ri. MED Cardiac Tissue Engineering Laboratory, Ri. MED Foundation, Palermo, IT
5. Department of Biological, Chemical and Pharmaceutical Science and Technologies, University of Palermo, Palermo, IT
6. Vascular Medicine Institute, Division of Cardiology, Department of Medicine, University of Pittsburgh, Pittsburgh, PA, US

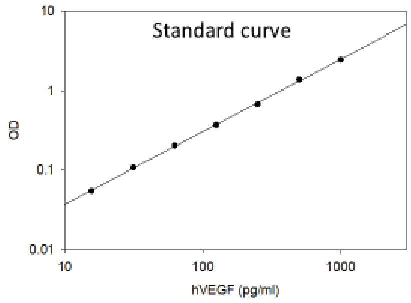

samples	VEGF Concentration (pg/ml)
High titer: 2.5*10^11 per well, 3 days	1260
Low titer: 2.5*10^10 per well, 3 days	733
Cells without AAV, 3 days	31
DMEM (no cells)	3

Figure S1.

In vitro transduction of HEK293 cells by AAV9-cmv-VEGF, quantified by ELISA. Cells were incubated with virus for 3 days. OD: optical density.
